# A predictive model for progression of CKD

**DOI:** 10.1097/MD.0000000000016186

**Published:** 2019-06-28

**Authors:** Hsueh-Lu Chang, Chia-Chao Wu, Shu-Pei Lee, Ying-Kai Chen, Wen Su, Sui-Lung Su

**Affiliations:** a School of Public Health; b School of Dentistry; c Center for General Education; d Personnel Officer, Tri-Service General Hospital; e Division of Nephrology, Department of Medicine, Tri-Service General Hospital, National Defense Medical Center; f Division of Nephrology, Department of Medicine, Zuoying Branch of Kaohsiung Armed Forces General Hospital, Kaohsiung; g Department of Nursing, Tri-Service General Hospital, National Defense Medical Center, Taipei, Taiwan, ROC.

**Keywords:** chronic kidney disease (CKD), end-stage renal disease (ESRD), predictive model, risk factors

## Abstract

The prevalence of chronic kidney disease (CKD) in Taiwan is 11.9%, and the incidence and prevalence of end-stage renal disease (ESRD) is ranked first in the world. The severity of CKD progression to ESRD is dependent on glomerular filtration rate and proteinuria. However, the risk factors for ESRD also include diabetes, hypertension, hyperlipidemia, age, sex, and so on, and predicting CKD progression using few variables is insufficient. Currently, there are no models with high accuracy and high explanatory power that could predict the risk of progression to dialysis in CKD patients in Taiwan. Our aim was to establish an optimal prediction model for CKD progression in patients

This study was a retrospective cohort study, which reviewed data from the “Public health insurance Pre-ESRD preventive program and patient health education program” that was implemented by the National Health Insurance Administration, Ministry of Health and Welfare. From 2006 to 2013, data of CKD patients from the Tri-Service General Hospital in Neihu District, Taipei City was examined. The data collected in this study included demographic variables, past medical history, and blood biochemical values. After exclusion of variables with >30% missing data, the remaining variables were interpolated using multiple imputations and inputted into the prediction model for analysis. The Cox proportion hazard model was used to investigate the influence of CKD risk factors on progression to dialysis. The strengths of various models were evaluated using likelihood ratios (LR), in order to identify a model which uses the least factors but has the strongest explanatory power.

The study results included 1549 CKD patients, of whom 1017 eventually had dialysis. This study found that in the prediction model with the best explanatory power, the influencing factors and hazard ratios (HR) were: age 0.95 (0.91–0.99), creatinine 1.03 (1.02–1.05), urea nitrogen 1.18 (1.14–1.23), and comorbid systemic diabetes 1.65 (1.45–1.88).

A prediction model was developed in this study, which could be used to carry out predictions based on blood biochemical values from patients, in order to accurately predict the risk of CKD progression to dialysis.

## Abstract 2

Supplemental Digital Content is available in the text

## 1 Introduction

Chronic kidney disease (CKD) is an important public health issue because CKD patients have an increased risk of end-stage renal disease (ESRD). Dialysis has charged the national health insurance system billions of dollars in recent years, and the costs are rising.^[1]^ The prevalence of CKD is around 14% in the USA and prevalence of ESRD is around 2043 per 1 million people, which is ranked third in the world.^[2]^ Besides Western countries, in Asian countries like Japan, the prevalence of adult CKD also reaches around 13.3% and the prevalence of ESRD is around 2411 patients per 1 million people,^[2,3]^ while even in China, the CKD prevalence rate is also around 10.8%.^[4]^ Furthermore, CKD patients also have poor cardiovascular outcomes and higher mortality rates.^[5]^ Hence, investigating the risk factors that could cause kidney function damage and deterioration, has become the highest priority in the prevention and treatment of kidney disease.

According to studies, the risk factors for CKD include sex, age, family medical history, obesity, smoking, high protein diet, anemia, proteinuria, and chronic diseases such as diabetes, hypertension, hyperlipidemia, metabolic diseases, cardiovascular diseases, and high uric acid, and so on.^[6]^ In the elderly age group (>65 years) with all 3 preexisting comorbidities of hypertension, hyperglycemia, and hyperlipidemia, the proportion of patients with pre-ESRD was as high as 18.3%, while the proportion of pre-ESRD was 5.2% in middle-age populations (40–64 years) with all 3 preexisting comorbidities of hypertension, hyperglycemia, and hyperlipidemia, for a difference of 3.5 times. Dialysis is an inevitable outcome if medication and diet were not timely used to control disease.^[7]^ On the other hand, there are not many prediction research studies on CKD disease progression.

The benefits of screening at-risk populations and estimating CKD progression are well established,^[8]^ and good risk prediction models are important for clinical practice, research, and public health policy. For clinical practice, the risk predictions could be used to triage patients for different management procedures.^[9]^ In addition, risk-treatment interactions are a major focus in clinical trials.^[10]^ Furthermore, high-risk patients may be identified early for public health interventions, which could improve the cost-effectiveness of ESRD prevention.^[5]^ Many studies have used these data to build prediction models.

This study will investigate the differences in CKD progression among patients and examine the relationship between biochemical test values and CKD disease progression, in order to establish a prediction model for CKD progression.

## 2 Materials and methods

### 2.1 Study population

The main experimental design of this study was a retrospective cohort study, which examined data from the “Public health insurance Pre-ESRD preventive program and patient health education program” that was implemented by the National Health Insurance Administration, Ministry of Health and Welfare. From 2006 to 2013, data of 2310 CKD patients from the Tri-Service General Hospital in Neihu District, Taipei City was reviewed, in order to investigate the characteristics of patients with Stages 1 to 5 of CKD. Patients with

(1) kidney transplant or dialysis,
(2) life expectancy less than 1 year,
(3) active metastatic malignancy,
(4) records without renal function data and
(5) congenital kidney disease was excluded from this study.

**Figure 1:**
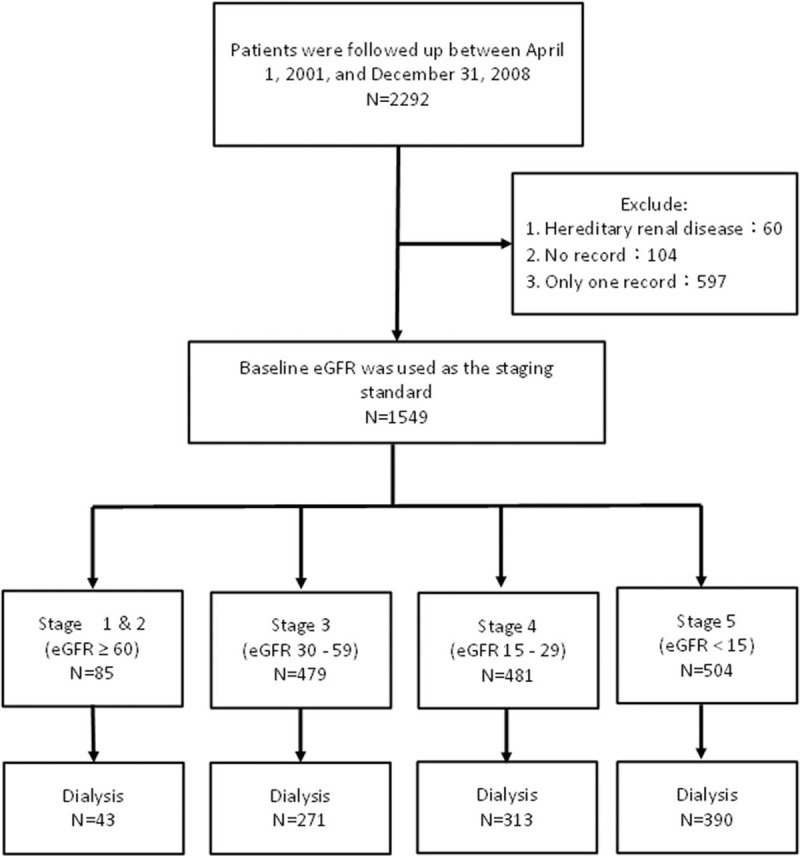
Flow diagram of the identification process for eligible studies.

Differences in disease progression between CKD patients were compared, and risk factors for kidney function deterioration in patients at various stages of CKD were identified. The time points when these risk factors exerted their effects on patients were retrospectively identified using glomerular filtration rate as an evaluation tool for kidney function deterioration. Patients who had transferred to other hospitals, on alternative therapies (traditional Chinese medicine or folk remedy), deaths, lost to follow-up, refused treatment or censored for other reasons were excluded. Finally, 1549 subjects were included in this study. Flow diagram of the identification process for eligible studies is shown in Figure 1. This study was reviewed and approved by the Tri-Service General Hospital committee on human research (Case number: 1-104-05-006).

### 2.2 Variables

#### 2.2.1 Dependent variable

The outcome of interest was kidney failure, which was defined by initiation of dialysis and censored for patients who had transferred to other hospitals, on alternative therapies (traditional Chinese medicine or folk remedy), deaths, lost to follow-up, refused treatment.

#### 2.2.2 Candidate predictive variables

The eGFR can be estimated using the CKD epidemiology collaboration creatinine equation^[11]^ 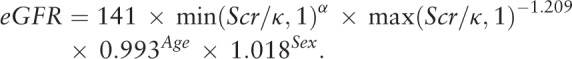


Here, *Scr* is the serum creatinine (mg/dL), and *Age* is the age of the patient (years). *Sex* is a dummy variable that is equal to 0 if male and 1 if female. Moreover, *κ* is equal to 0.7 if female and 0.9 if male, and *α* is equal to −0.329 if female and −0.411 if male. The above equation was shown to perform better than an earlier version.^[11,12]^

Based on relevant factors, the characteristics of CKD patients were classified into 4 main categories:

(1) Basic demographics such as sex, age, body mass index (BMI), family medical history, primary disease categories;
(2) Risk factors such as hypertension, hyperlipidemia, hyperglycemia, proteinuria, and hypoproteinemia;
(3) Systemic co-morbidity such as diabetes, congestive heart failure, malignant tumors, ischemic heart disease, cerebrovascular disease, anemia associated with chronic liver disease/cirrhosis, tuberculosis, neuropathy, retinopathy, autoimmune disease, and so on
(4) Basic biochemical test values.

### 2.3 Statistical analysis

Any variables with a large missing rate (>30%) will be removed.^[13]^ Variables with skewed distributions will be log transformed because of the subsequent multiple imputation step. Rubin presented a method for combining analysis results from *m* imputation profiles.^[14]^*Q*_i_ and *U*_i_ are the coefficient of interest and its variance for each imputation profile (I = 1, 2, 3,…, m), respectively. The overall coefficient (*Q*), overall variance (*U*) and their degrees of freedom (*df*) can be calculated using  
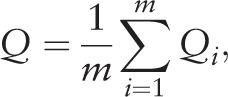



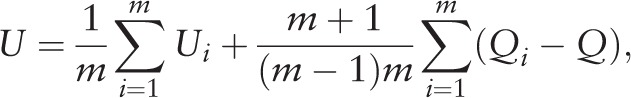


and 
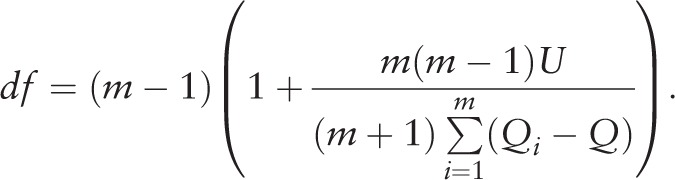


This method can also be used for significance testing. If the null hypothesis is set to *Q* = 0, then we can test *Q* using 
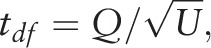


which is based on the t-distribution.

For analysis of continuous variables, *t* tests were used to evaluate continuous variables with mean values and standard deviations, while variables that do not follow the normal distribution will be analyzed using the Kruskal–Wallis test for significant differences. For analysis of discrete variables that were presented as percentages, the Chi-square test was used to verify if there were significant differences in 2 categorical variables.

Comparison of basic data for CKD patients was then carried out. This data included sex, age, BMI, others, and family medical history such as diabetes, hypertension, heart disease, cerebrovascular disease, hyperlipidemia, kidney disease, congenital kidney disease, polycystic kidney disease, gout, and so on. Primary disease categories were divided into renal parenchymal disease, systemic disease, obstructive nephropathy and diseases of the urinary system, renal vascular disease, genetic disease, other renal failure with known causes, idiopathic renal failure, and so on.

Finally, the Cox Proportion Hazard Model survival analysis was used to investigate the risks of CKD progression to dialysis. This analysis included:

(1) Estimation of survival rate, which describes the number of CKD patients who will require dialysis treatment after follow-up for this period of time and time points of progression at various stages of CKD;
(2) Survival analysis of multiple risk factors in order to understand whether each risk factor has an effect on progression to dialysis.

Besides survival time, this includes many other risk factors such as basic demographic characteristics (sex, age, BMI, family medical history, primary disease category) and patient condition (hypertension, hyperlipidemia, hyperglycemia, proteinuria, hypoproteinemia), and so on. information. Cox regression analysis was used to process these risk factors.

Model performance comparison was based on Likelihood Ratios (LR) of each model. The LR is following Chi-square distribution, so we test the model performance by Chi-square test. Significant level was set as 0.05, and all analysis was performed by R language (v.3.2.3).

## 3 Results

### 3.1 Study population description

All CKD patients were classified according to disease stages and described. As the number of patients in stages 1 and 2 were comparatively fewer, patients in these stages were combined. There were 85 cases in stages 1 and 2, 479 cases in stage 3, 481 cases in stage 4 and 504 cases in stage 5.

Table 1 shows the differences in basic demographic variables between the various stages. We found that age, male sex, proportion of dialysis in various CKD stages, days of follow-up, systolic pressure, diastolic pressure, and glomerular filtration rate showed statistically significant differences.

**Table 1:**
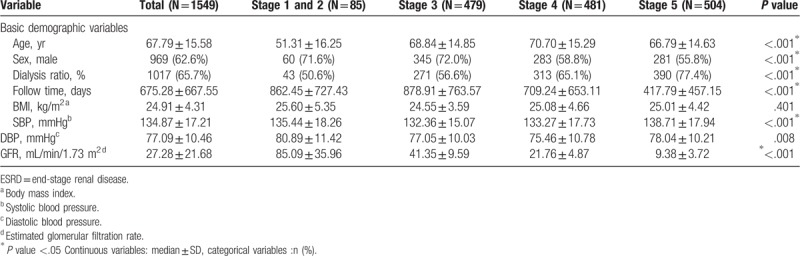
Characteristics of basic demographic variables among stage 1 to 5 of ESRD.

The distribution of biochemical test values of patients at various stages is shown in Table 2. Among them, 13 biochemical test values such as erythrocyte, hemoglobin, hematocrit, urea nitrogen, creatinine, albumin, sodium, calcium, cholesterol, triglycerides, fasting blood glucose, GOT, and GPT showed statistically significant differences between the various stages.

**Table 2:**
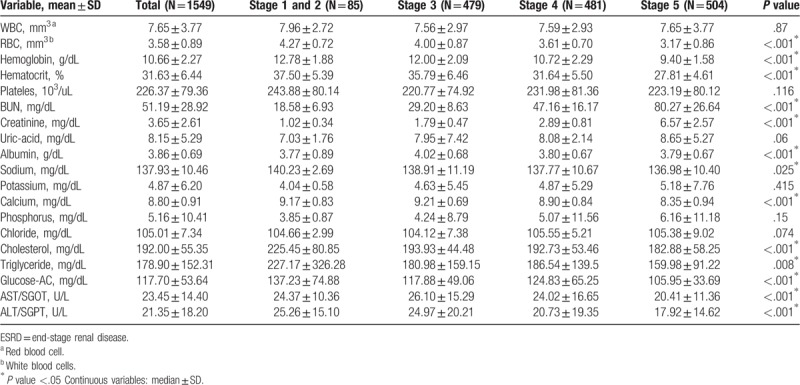
Characteristics of blood biochemical value among stage 1 to 5 of ESRD.

Tables 3 and 4 respectively describe the distribution of comorbid systemic diseases and family medical history in CKD patients in various stages. Five comorbid systemic diseases (diabetes, congestive heart failure, cerebrovascular disease, hyperlipidemia, and anemia) showed significant differences between the various stages. There were no statistically significant differences in family medical history between the various stages.

**Table 3:**
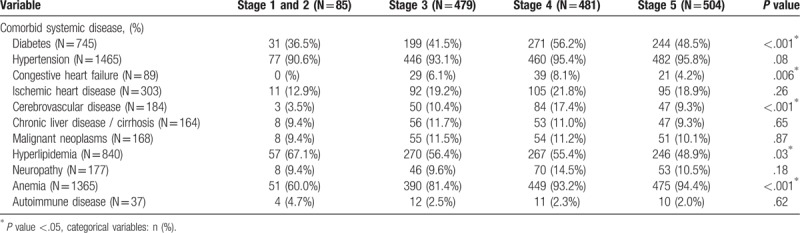
The distribution of systemic disease in each stage of chronic kidney disease.

**Table 4:**
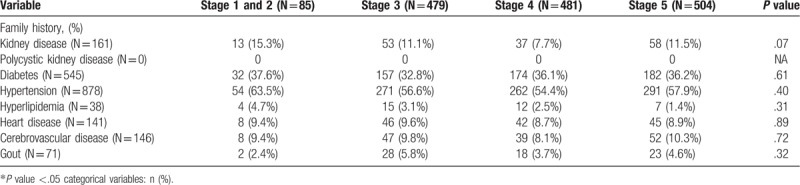
Distribution of family history of each stage of chronic kidney disease.

### 3.2 Prediction model performance in the study population

#### 3.2.1 Univariate analysis

Table 5 shows the single variable analysis of basic demographic variables and biochemical test values. For every 10 year increase in age, the risk of ESRD will decrease significantly, hazard ratios (HR) 0.95 (95% confidence interval 0.91–0.99). For systolic pressure, every increase in 10 mmHg significantly increases the risk of ESRD, HR 1.11 (1.06–1.16). For eGFR, every increase of 5 mL/min/1.73 m^2^ will significantly decrease the risk of ESRD, HR 0.88 (0.86–0.90). For biochemical test values, when all other conditions are equal, every 10^6^/uL increase in erythrocyte count will significantly decrease the risk of ESRD, HR 0.74 (0.67–0.80). For hemoglobin, every 1 g/dL increase in hemoglobin will significantly decrease the risk of ESRD, HR 0.84 (0.82–0.87). For hematocrit, every 5% increase will decrease the risk of ESRD, HR 0.78 (0.74–0.82). For platelet count, every 10^4^/uL increase will significantly decrease the risk of ESRD, HR 0.98 (0.97–1.00). For urea nitrogen, every 5 mg/dL increase will significantly increase the risk of ESRD, HR 1.10 (1.09–1.11). For creatinine, every increase of 1 mg/dL will significantly increase the risk of ESRD, HR 1.24 (1.22–1.27). For albumin, every 1 g/dL increase will significantly increase the risk of ESRD, HR 1.06 (1.03–1.10). For calcium, every 1 meq/L increase will significantly decrease the risk of ESRD, HR 0.67 (0.61–0.73). For GOT, every increase of 5U/L will significantly decrease the risk of ESRD, HR 0.94 (0.90–0.97). For GPT, every increase of 5U/L will significantly decrease the risk of ESRD, HR 0.97 (0.94–0.99).

**Table 5:**
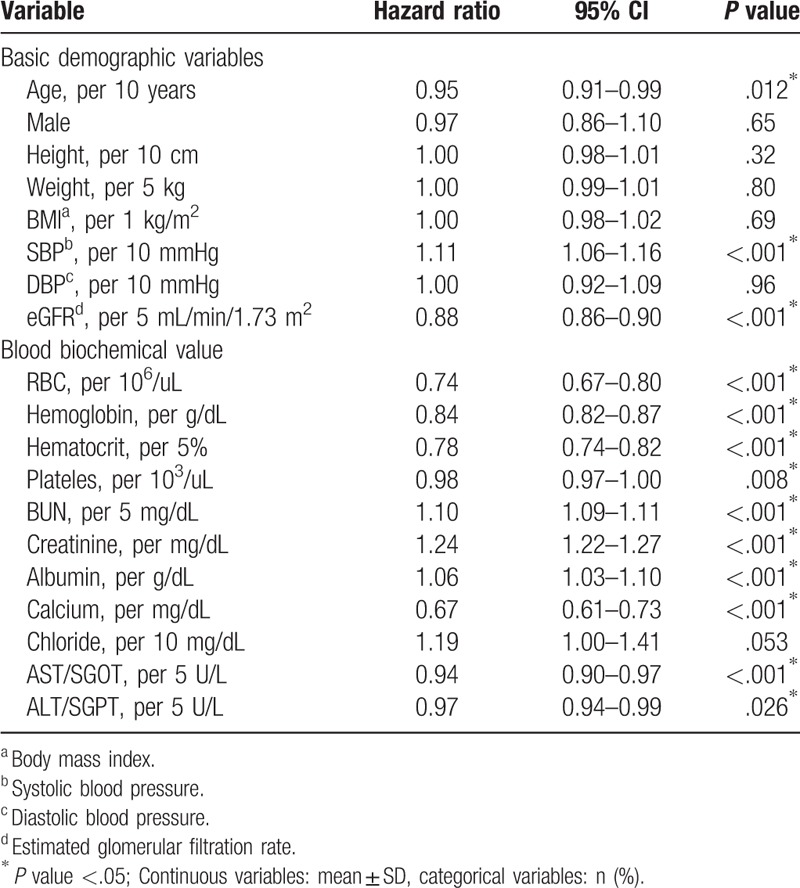
Univariate analysis of factors affecting the development of chronic kidney disease to dialysis.

In Table 6, the influence of comorbid systemic disease on progression to ESRD was investigated. Patients with diabetes will have a higher chance of progression to dialysis, (HR 1.47, 95% CI: 1.28–1.64) when compared with patients without. Patients with hypertension have a higher chance of progression to dialysis, (HR 1.52, 95% CI: 1.13–2.05) when compared with patients without. Patients with chronic liver disease/cirrhosis have a lower chance of progression to dialysis, HR 0.74 (0.61–0.91) when compared with patients without. Patients with hyperlipidemia have a lower chance of progression to dialysis, HR 0.78 (0.69–0.88) when compared with patients without. Patients with anemia have a higher chance of progression to dialysis, HR 1.67 (1.35–2.06) when compared with patients without.

**Table 6:**
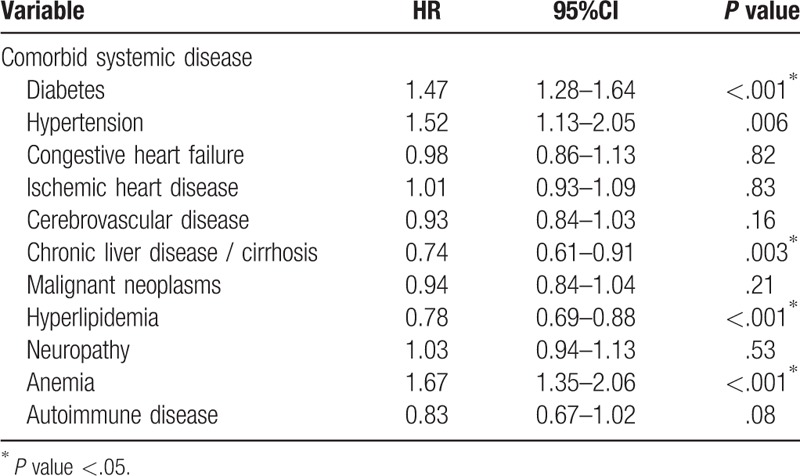
Univariate analysis of the associated systemic disease affecting the progression of chronic kidney disease to dialysis.

In Table 7, the influence of family medical history on progression to ESRD was investigated. When all other conditions are equal, patients with a family history of diabetes have a higher chance of progression to dialysis, HR 1.25 (1.10–1.43) when compared with patients without. Patients with a family history of hypertension have a higher chance of progression to dialysis, HR 1.19 (1.03–1.35) when compared with patients without. Tables 5 to 7 show the effects of various independent variables on CKD progression. The detailed stratified analysis of each CKD stage based on Tables 5 to 7 were shown in Supplementary (see Supplemental Content, which illustrates detailed stratified analysis of each CKD stage): the *P* value of heterogeneity shows statistically significant differences at the various independent variables include male, SBP, eGFR, creatinine, albumin, calcium, hyperlipidemia, and autoimmune disease. However, these do not affect our results.

**Table 7:**
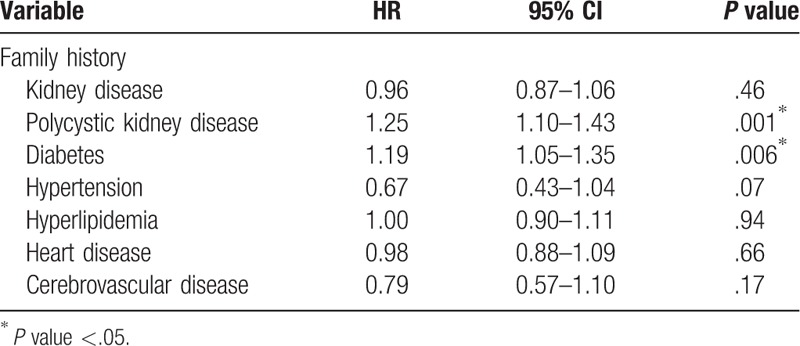
Univariate analysis of family history of patients with chronic kidney disease progressing to dialysis.

#### 3.2.2 Multivariate analysis

In this study, after all cases were entered into the dialysis risk prediction model, some variables showed collinearity and an exhaustive method were used for variable selection. Table 8 presented the important models during the process. Among the 2-factor models, the model with the best predictive power was Model 1 (LR = 153.046), which used age and glomerular filtration rate. Among the 3-factor models which used age and glomerular filtration rate as bases, the best 2 models were Model 2 (LR = 204.652) and Model 3 (LR = 354.422), which respectively added hemoglobin and urea nitrogen. However, an exhaustive search of all our 3-factor models found that among 3-factor models that used age, hemoglobin and creatinine, the model with the best predictive power was Model 4 (LR = 434.734). Following that, we found Models 5 and 6 to be the best models for 4-factor and 5-factor models, respectively. However, we found that the 4-factor combination (LR = 479.391) had a higher explanatory power than the 5-factor combination (LR = 449.927) and all 3-factor combinations, hence Model 5 was selected as the final prediction model. The factors and effects in this model are: age (every increase of 10 years), HR 0.95 (0.91–0.99); urea nitrogen (every increase of 5 mg/dL), HR 1.03 (1.02–1.05); creatinine (every increase of 1 mg/dL), HR 1.18 (1.14–1.23) and comorbid systemic diabetes, HR 1.65 (1.45–1.88).

**Table 8:**
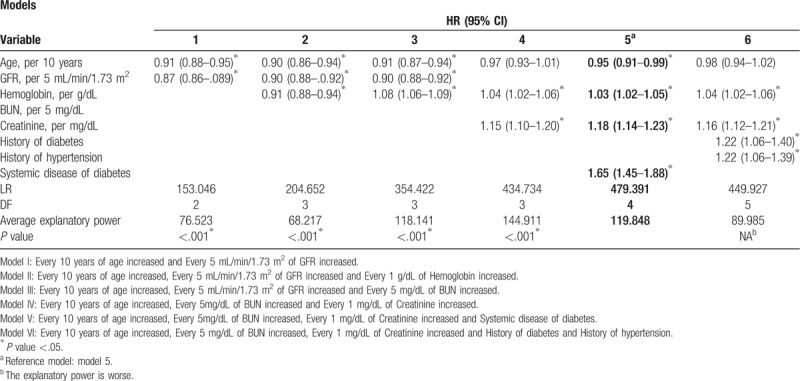
Multivariate analysis of the factors affecting the progression of chronic kidney disease to dialysis.

## 4 Discussion

This study developed a prediction model which found that the risk factors that increase CKD progression to dialysis include decrease in age, increase in urea nitrogen, increase in creatinine, and presence of comorbid systemic diabetes. All these factors could cause kidney function deterioration and accelerate the time to progression to dialysis.

Regarding age as a risk factor, our study found that in 1549 CKD patients, every 10 year rise in age will decrease the risk of dialysis (HR: 0.95, 95% CI: 0.91–0.99), which was similar to a 2013 Taiwan study by Lin ChunMei et al (HR: 0.99; 95% CI: 0.98–0.99).^[15]^ Overseas studies have also found that as patient age increases, the risk of death also correspondingly increases, resulting in the phenomena of decrease in risk of dialysis (HR 0.82).^[16]^

This study found that in CKD patients, every 5 mg/dL increase in urea nitrogen will increase the overall risk of dialysis by 1.03 times (95% CI: 1.02–1.05). This was similar to a 2011 study in the US and Canada on Caucasians and other races that found that persistent elevation of urea nitrogen will increase the risk of death, and elevated urea nitrogen values was a risk factor accelerating CKD progression to dialysis and death.^[17]^

With regards to comorbid systemic disease and family medical history, our study found that CKD patients with diabetes had a 1.65 times increase (95% CI: 1.45–1.88) in progression to dialysis, compared with patients without diabetes. Diabetes has previously been shown in many studies to increase the risk of CKD progression to dialysis. In 2010, a study from US found that diabetes will increase the risk of dialysis (HR 1.74, 95% CI: 0.95–3.19), and even though there was no statistical significance, but this was still found to be a high-risk phenomenon.^[17]^ In 2012, a prospective study in the US found that in CKD patients, presence of diabetes increases the risk of progression to dialysis by 1.57 times (95% CI: 1.29–1.92).^[18]^

Based on the prediction model constructed in this study, the risk factors in the model with the highest explanatory power for CKD progression to dialysis include age (every increase of 10 years), urea nitrogen (every increase of 5 mg/dL), creatinine (every increase of 1 mg/dL) and comorbid systemic diabetes. In a 2011 study from Canada on an ESRD prediction model for CKD patients, the constructed model included age, sex, systolic pressure, diastolic pressure, albumin, phosphorous, calcium, hypertension, and diabetes.^[13]^

In this study, age (every increase of 10 years) could reduce the risk of dialysis by 0.95 times (95% CI: 0.91–0.99) while in the Canadian study, it was 0.82 times. Every 1 mg/dL increase in creatinine will increase the risk of dialysis by 1.18 times (95% CI: 1.14–1.23). Creatinine in the blood mainly arises as metabolic products of muscle activity and is excreted daily in the urine by the kidneys. If there are problems in kidney function, daily creatinine produced would not be completely excreted, resulting in an increase in blood creatinine concentration. The biggest difference observed in our study was the presence of diabetes as we found that comorbid diabetes will increase the risk of dialysis by 1.65 times (95% CI: 1.45–1.88) while the Canadian study showed that comorbid diabetes will reduce the risk of dialysis by 0.89 times.^[13]^

One limitation of this study was that the patients were not treated by a single physician, and different physicians have different habits of conducting biochemical tests, resulting in large omissions of biochemical test values in the data. To avoid affecting the accuracy of the deduction, biochemical values that had more than 30% missing were not included in the analysis. In addition, there was a lot of missing data during collection; hence this study uses multiple interpolation methods to make up for the missing data. Even though the interpolated data may not be completely accurate, but at least all patient data could be used for analysis.

## 5 Conclusions

Based on results from this study, we developed a prediction model that can be used to predict the probability of progression to dialysis in CKD patients and suggest future interventions to prevent the progression of CKD such as strict control of creatinine, urea nitrogen, albumin, hemoglobin, calcium, and other related biochemical test values. If patients have hypertension or diabetes as comorbidities, greater attention must be paid on deterioration of kidney function in order to reduce the occurrence of dialysis.

In this study, there were missing biochemical data, hence we suggest collecting complete and appropriate biochemical test values in future studies on CKD patients undergoing dialysis, which could lead to further discoveries on the influence of biochemical test values.

## Author contributions

**Conceptualization:** Ying-Kai Chen.

**Writing – original draft:** Hsueh-Lu Chang, Chia-Chao Wu, Shu-Pei Lee, Ying-Kai Chen, Wen Su, Sui-Lung Su.

Conceived and designed the experiments: Hsueh-Lu Chang, Chia-Chao Wu, Sui-Lung Su.

Performed the experiments: Hsueh-Lu Chang, Shu-Pei Lee, Ying-Kai Chen, Wen Su.

Analyzed the data: Hsueh-Lu Chang, Shu-Pei Lee.

Contributed reagents/materials/analysis tools: Hsueh-Lu Chang, Sui-Lung Su.

Wrote the manuscript: Hsueh-Lu Chang.

Prepared the figures and/or tables: Hsueh-Lu Chang, Chia-Chao Wu.

Reviewed drafts of the paper: Hsueh-Lu Chang, Chia-Chao Wu, Sui-Lung Su.

## Supplementary Material

Supplemental Digital Content

## References

[1] Go AS Chertow GM Fan D Chronic kidney disease and the risks of death, cardiovascular events, and hospitalization. N Engl J Med 2004;351:1296–305. 15385656 10.1056/NEJMoa041031

[2] *The 2015 Annual Data Report*. United States Renal Data System, 2015 p13–14.

[3] Imai E Horio M Watanabe T Prevalence of chronic kidney disease in the Japanese general population. Clin Exp Nephrol 2009;13:621–30. 19513802 10.1007/s10157-009-0199-x

[4] Zhang L Wang F Wang L Prevalence of chronic kidney disease in China: a cross-sectional survey. Lancet 2012;379:815–22. 22386035 10.1016/S0140-6736(12)60033-6

[5] Manns B Hemmelgarn B Tonelli M Population based screening for chronic kidney disease: cost effectiveness study. BMJ 2010;341:58–69. 10.1136/bmj.c5869 PMC2975430 21059726

[6] *2014 Annual Report on Kidney Disease in Taiwan*, National Health Research Institutes, 2014. p. 31–33.

[7] Kuo HW Tsai SS Tiao MM Epidemiological features of CKD in Taiwan. Am J Kidney Dis 2007;49:46–55. 17185145 10.1053/j.ajkd.2006.10.007

[8] Li PK Weening JJ Dirks J A report with consensus statements of the International Society of Nephrology 2004 Consensus Workshop on Prevention of Progression of Renal Disease, Hong Kong, June 29, 2004. Kidney Int Suppl 2005;S2–7. 10.1111/j.1523-1755.2005.09401.x 15752234

[9] Leslie WD Morin S Lix LM A before-and-after study of fracture risk reporting and osteoporosis treatment initiation. Ann Intern Med 2010;153:580–6. 21041577 10.7326/0003-4819-153-9-201011020-00007

[10] Kent DM Hayward RA Limitations of applying summary results of clinical trials to individual patients: the need for risk stratification. JAMA 2007;298:1209–12. 17848656 10.1001/jama.298.10.1209

[11] Levey AS Stevens LA Schmid CH A new equation to estimate glomerular filtration rate. Ann Intern Med 2009;150:604–12. 19414839 10.7326/0003-4819-150-9-200905050-00006 PMC2763564

[12] Levey AS Bosch JP Lewis JB A more accurate method to estimate glomerular filtration rate from serum creatinine: a new prediction equation. Modification of Diet in Renal Disease Study Group. Ann Intern Med 1999;130:461–70. 10075613 10.7326/0003-4819-130-6-199903160-00002

[13] Tangri N Stevens LA Griffith J A predictive model for progression of chronic kidney disease to kidney failure. JAMA 2011;305:1553–9. 21482743 10.1001/jama.2011.451

[14] Rubin DB Schenker N Multiple imputation for interval estimation from simple random samples with ignorable nonresponse. J Am Stat Assoc 1986;81:366–74.

[15] Lin CM Yang MC Hwang SJ Progression of stages 3b-5 chronic kidney disease--preliminary results of Taiwan national pre-ESRD disease management program in Southern Taiwan. J Formos Med Assoc 2013;112:773–82. 24309170 10.1016/j.jfma.2013.10.021

[16] Pencina MJ D’Agostino RB Sr D’Agostino RB Jr Evaluating the added predictive ability of a new marker: from area under the ROC curve to reclassification and beyond. Stat Med 2008;27:157–72. 17569110 10.1002/sim.2929

[17] Desai AS Toto R Jarolim P Association between cardiac biomarkers and the development of ESRD in patients with type 2 diabetes mellitus, anemia, and CKD. Am J Kidney Dis 2011;58:717–28. 21820220 10.1053/j.ajkd.2011.05.020

[18] Jardine MJ Hata J Woodward M Prediction of kidney-related outcomes in patients with type 2 diabetes. Am J Kidney Dis 2012;60:770–8. 22694950 10.1053/j.ajkd.2012.04.025

