# Supplementary material for: A predictive model for progression of CKD
Source: Medicine (Baltimore). 2019 Jun 28;98(26):e16186. doi: 10.1097/MD.0000000000016186 (PMC6617424; doi:10.1097/MD.0000000000016186)
Supplement: Supplemental Digital Content [file medi-98-e16186-s001.pdf]

Supplementary table1、Patient characteristics by baseline stage of CKD

variable	stage1、2			stage3			stage4			stage5			Heterogeneity P-value
	HR	95% CI	P	HR	95% CI	P	HR	95% CI	P	HR	95% CI	P	
Basic demographic variables													
Age, per 10 years	0.80	0.65-1.00	0.04*	0.96	0.89-1.04	0.29	0.92	0.86-0.99	0.03*	0.93	0.87-1.00	0.06	0.46
Male	0.58	0.32-1.07	0.08	0.88	0.68-1.14	0.34	1.31	1.04-1.65	0.02*	1.31	1.07-1.60	0.01*	0.008*
Height, per 10 cm	1.00	0.95-1.05	0.98	1.00	0.98-1.02	0.94	1.00	0.98-1.02	0.65	1.01	0.99-1.02	0.49	0.87
Weight , per 5 kg	0.98	0.95-1.01	0.14	1.00	0.98-1.01	0.79	1.01	1.00-1.02	0.39	1.00	0.99-1.01	0.83	0.39
BMI[a], per 1 kg/m^2	0.90	0.81-1.01	0.067	0.99	0.95-1.04	0.77	1.02	0.99-1.06	0.21	1.00	0.97-1.03	0.94	0.17
SBP[b], per 10 mmHg	0.95	0.74-1.23	0.72	1.05	0.94-1.17	0.39	1.04	0.95-1.15	0.38	1.16	1.07-1.25	<0.001*	<0.001*
DBP[c], per 10 mmHg	0.91	0.60-1.37	0.65	0.96	0.82-1.13	0.65	1.01	0.87-1.17	0.89	1.05	0.92-1.20	0.45	0.81
eGFR[d], per 5 mL/min/1.73 m^2	1.02	1.00 - 1.03	0.037*	0.97	0.96 - 0.99	0.001*	0.96	0.93-0.98	0.001*	0.90	0.86-0.93	<0.001*	<0.001*

1.*：P-value < 0.05 indicating statistical significance.

2.Heterogeneity P-value < 0.05 ：indicating statistical significance. between CKD stage

Supplementary table2、 Patient biochemical measures by baseline stage of CKD

variable	stage1、2			stage3			stage4			stage5			Heterogeneity P-value
	HR	95% CI	P	HR	95% CI	P	HR	95% CI	P	HR	95% CI	P	
Blood biochemical value													
RBC, per 10^6/uL	0.62	0.37-1.05	0.077	0.82	0.68-1.00	0.048*	0.92	0.76-1.12	0.41	0.97	0.84-1.11	0.63	0.29
Hemoglobin, per g/dl	0.80	0.66-0.97	0.026*	0.89	0.83-0.95	0.001*	0.90	0.84-0.96	0.002*	0.92	0.86-0.99	0.027*	0.59
Hematocrit, per 5%	0.93	0.87-1.00	0.03*	0.98	0.96-1.00	0.026*	0.97	0.95-0.99	0.008*	0.97	0.95-0.99	0.011*	0.59
Plateles, per 10^3/uL	1.00	0.99-1.00	0.54	1.00	0.99-1.00	0.31	1.00	0.99-1.01	0.54	0.99	0.99-1.00	0.004*	0.64
BUN, per 5 mg/dl	1.00	0.95-1.05	0.97	1.02	1.00-1.03	0.025*	1.01	1.00-1.02	0.036*	1.01	1.01-1.02	<0.001*	0.65
Creatinine, per mg/dL	0.35	0.14-0.86	0.021*	1.07	0.82-1.40	0.63	1.30	1.14-1.50	<0.001*	1.17	1.12-1.21	<0.001*	0.02*
Albumin, per g/dL	1.02	0.86-1.20	0.86	1.10	1.04-1.17	0.001*	1.06	1.00-1.12	0.06	0.97	0.92-1.03	0.31	0.02*
Calcium, per mg/dL	0.36	0.20-0.65	0.001*	0.99	0.77-1.29	0.95	0.88	0.75-1.04	0.88	0.67	0.58-0.77	<0.001*	0.01*
Chloride, per 10 mg/dL	0.99	0.89-1.09	0.86	1.04	0.99-1.08	0.15	1.02	0.99-1.06	0.16	1.00	0.98-1.02	0.70	0.30
AST/SGOT, per 5 U/L	0.98	0.95-1.01	0.21	1.00	0.98-1.02	0.96	0.99	0.98-1.00	0.12	1.00	0.99-1.01	0.56	0.57
ALT/SGPT, per 5 U/L	0.99	0.97-1.01	0.13	1.00	0.99-1.01	0.99	1.00	0.99-1.01	0.88	1.00	0.99-1.01	0.80	0.52

1.*：P-value < 0.05 indicating statistical significance.

2.Heterogeneity P-value < 0.05 ：indicating statistical significance. between CKD stage

Supplementary table3、 Patient comorbidity by baseline stage of CKD

variable	stage1、2			stage3			stage4			stage5			Heterogeneity P-value
	HR	95% CI	P	HR	95% CI	P	HR	95% CI	P	HR	95% CI	P	
Comorbid systemic disease													
Diabetes	1.03	0.54-1.95	0.94	1.28	1.00-1.65	0.05	1.40	1.11-1.76	0.01*	1.67	1.36-2.05	0.01*	0.26
Hypertension	1.07	0.25-4.49	0.93	1.18	0.73-1.89	0.49	1.47	0.86-2.52	0.16	1.72	0.92-3.23	0.09	0.78
Congestive heart failure	0.83	0.36-1.91	0.66	0.87	0.63-1.20	0.39	0.97	0.74-1.26	0.81	1.18	0.92-1.51	0.19	0.44
Cerebrovascular disease	1.94	0.45-8.34	0.37	0.77	0.50-1.17	0.22	0.92	0.68-1.25	0.58	0.88	0.62-1.27	0.51	0.65
Chronic liver disease / cirrhosis	0.54	0.21-1.41	0.21	0.63	0.43-0.93	0.02*	0.76	0.53-1.09	0.13	0.89	0.64-1.24	0.48	0.52
Malignant neoplasms	1.21	0.37-4.02	0.75	0.92	0.63-1.35	0.68	0.83	0.57-1.20	0.32	0.95	0.67-1.34	0.76	0.91
Hyperlipidemia	0.51	0.26-1.01	0.05	0.72	0.56-0.92	0.01*	0.79	0.63-0.99	0.04*	1.06	0.87-1.29	0.58	0.03*
Neuropathy	0.88	0.34-2.27	0.78	0.90	0.59-1.38	0.63	1.20	0.89-1.63	0.23	1.07	0.78-1.47	0.67	0.71
Anemia	1.19	0.64-2.22	0.58	1.51	1.09-2.11	0.01*	1.10	0.67-1.83	0.70	1.12	0.70-1.78	0.64	0.63
Autoimmune disease	4.22	1.26-14.2	0.02*	0.60	0.28-1.28	0.19	0.71	0.31-1.59	0.40	0.56	0.25-1.26	0.16	0.03*

1.*：P-value < 0.05 indicating statistical significance.

2.Heterogeneity P-value < 0.05 ：indicating statistical significance. between CKD stage

Supplementary table4、Patient family history by baseline stage of CKD

variable	stage1、2			stage3			stage4			stage5			Heterogeneity P-value
	HR	95% CI	P	HR	95% CI	P	HR	95% CI	P	HR	95% CI	P	
Family history													
Kidney disease	0.54	0.16-1.77	0.31	1.11	0.78-1.58	0.56	0.88	0.59-1.31	0.53	0.98	0.71-1.35	0.91	0.62
Diabetes	1.48	0.75-2.91	0.26	1.01	0.78-1.31	0.95	1.40	1.12-1.75	0.004*	1.30	1.06-1.59	0.01*	0.26
Hypertension	0.98	0.52-1.83	0.94	1.31	1.02-1.68	0.04*	1.13	0.90-1.42	0.29	1.22	1.00-1.50	0.05	0.76
Hyperlipidemia	4.49	1.03-19.65	0.05	0.66	0.33-1.34	0.25	0.59	0.24-1.43	0.24	1.09	0.45-2.64	0.84	0.09
Heart disease	0.99	0.38-2.58	0.98	0.99	0.65-1.51	0.96	1.16	0.80-1.69	0.42	0.82	0.57-1.17	0.28	0.61
Cerebrovascular disease	0.59	0.18-1.94	0.39	0.90	0.58-1.41	0.64	0.97	0.66-1.43	0.87	0.89	0.64-1.22	0.45	0.89

1.*：P-value < 0.05 indicating statistical significance.

2.Heterogeneity P-value < 0.05 ：indicating statistical significance. between CKD stage
